# Utility of Urinary Nephrin in Patients With and Without Diabetic Nephropathy and Its Correlation With Albuminuria

**DOI:** 10.7759/cureus.20102

**Published:** 2021-12-02

**Authors:** Manupati Surya, Medha Rajappa, Vadivelan M

**Affiliations:** 1 Medicine, Jawaharlal Institute of Postgraduate Medical Education and Research, Puducherry, IND; 2 Biochemistry, Jawaharlal Institute of Postgraduate Medical Education and Research, Puducherry, IND

**Keywords:** podocyte-specific protein, type 2 diabetes, diabetic nephropathy, albuminuria, urinary nephrin

## Abstract

Introduction

Nephrin is a podocyte-specific protein that may serve as a urinary biomarker in patients with diabetic nephropathy. The objectives of the study were to determine urinary nephrin levels in patients with and without type 2 diabetic nephropathy and to assess the correlation between urinary nephrin and albuminuria.

Methods

This was a cross-sectional comparative study that was carried out at Jawaharlal Institute of Postgraduate Medical Education and Research (JIPMER), Puducherry over 18 months. Diabetic study subjects were divided into three groups-normoalbuminuria, microalbuminuria, and macroalbuminuria. Urinary albumin was detected by the dipstick method in a spot urine sample for all study subjects. In subjects with trace or no albuminuria, nephelometry was used to quantify urinary albumin levels. Urinary nephrin was estimated by the ELISA technique for all study subjects.

Results

Subjects in the microalbuminuria group had higher urinary nephrin levels than those in the normoalbuminuria and macroalbuminuria groups. There was a weak positive correlation between urinary albumin and nephrin levels among the study subjects (p < 0.001).

Conclusion

Urinary nephrin levels are increased in patients with diabetic nephropathy. There was a weak positive correlation between urinary albumin and nephrin levels among these patients.

## Introduction

Diabetic nephropathy is a common microvascular complication in patients with diabetes mellitus next to neuropathy [1]. Diabetic nephropathy occurs in 30-40% of patients. It gradually progresses to end-stage renal disease (ESRD) over a period of 10-25 years [2].

Diabetic nephropathy causes pathologic alterations in structural and functional components of kidneys due to hyperfiltration glomerular injury. In patients with type 2 diabetes mellitus, renal morphologic changes are more heterogeneous, and diabetic glomerulopathy is less severe than in patients with type 1 diabetes mellitus with similar urinary albumin levels. Persistent albuminuria is the hallmark of diabetic nephropathy in patients with type 1 and type 2 diabetes mellitus [3].

Microalbuminuria is currently the gold standard for the diagnosis of diabetic nephropathy. However, it does not correlate with the progression of renal failure and can be caused by other conditions like exercise, urinary tract infections, and congestive cardiac failure among others. Hence, newer biomarkers have been proposed to quantitatively and qualitatively correlate with the progression of diabetic nephropathy [4].

Podocyte-specific proteins like nephrin, podocin, and synaptopodin are currently being investigated as biomarkers for diabetic nephropathy. Podocyte is a specialized visceral epithelial cell that maintains the renal filtration barrier along with the glomerular basement membrane (GBM) and endothelial cell layer, which prevents urinary loss of protein. In diabetic nephropathy, the expression of nephrin is significantly reduced on podocytes. Also, phenotypic alteration of podocytes occurs in diabetic nephropathy. This leads to disruption of the renal filtration barrier causing urinary loss of nephrin [4].

There are only a few Indian studies to date that have been done to assess the correlation of urinary nephrin and albuminuria in patients with diabetic nephropathy.

The objectives of the study were to estimate urinary nephrin levels in patients with and without type 2 diabetic nephropathy and to assess the correlation between urinary nephrin and albuminuria in patients with type 2 diabetic nephropathy.

## Materials and methods

Study design

This was a cross-sectional comparative study that recruited patients attending the Medicine out-patient department (OPD) and Diabetes clinic of Jawaharlal Institute of Postgraduate Medical Education and Research (JIPMER), Puducherry from March 2019 to October 2020.

Inclusion & exclusion criteria

Patients with type 2 diabetes mellitus on oral hypoglycemic drugs or insulin were included as the study subjects. Patients with urinary tract infection, those on angiotensin-converting enzyme (ACE) inhibitors or angiotensin receptor blockers (ARBs) and having serum creatinine value more than 1.5 mg/dL (males) & more than 1.4 mg/dL (females) were excluded from the study.

Data collection

Three groups were formed for this study. Group A included diabetic patients without albuminuria (urinary albumin-creatinine ratio (UACR) 0-30 mg/g of creatinine), group B included patients with microalbuminuria (UACR 30-300 mg/g of creatinine), and group C included patients with macroalbuminuria (UACR > 300 mg/g of creatinine).

Based on a previous study done by Jim et al., the sample size was calculated using the formula, n = (1.96 × SD/D)2 where n is the sample size, SD is the standard deviation from the study and D is the absolute precision. With a 95% confidence interval (CI) and 20% relative precision, the sample size was calculated to be 4900. Since it was impossible to reach the number with available resources, 147 subjects were recruited for the study. They were divided into three groups: 1) diabetic subjects with normoalbuminuria (n = 65), 2) microalbuminuria (n = 45) and 3) macroalbuminuria (n = 37).

The study was approved by the Institute Ethics Committee (Human Studies) of Jawaharlal Institute of Postgraduate Medical Education and Research (JIPMER), Puducherry before its commencement (Approval number-JIP/IEC/2019/083 dated 09/05/2019). All subjects gave informed, written consent for participation in the study. Data was obtained from the study subjects and their health records regarding the duration of diabetes. Height, weight, and body mass index were recorded for all the subjects. A random urine sample was collected from all the subjects. Urine examination for albumin was done by using a simple and rapid dipstick test. Subjects were then categorized as dipstick positive and dipstick negative groups. Subjects with dipstick positive albuminuria were included in the macroalbuminuria group. All urine samples (dipstick positive and negative) were centrifuged and 2 mL each of the supernatant fluid was collected in two separate Eppendorf tubes for quantitative estimation of albumin and nephrin. All urine samples were stored at -20°C in the deep freezer.

Urinary albumin level was estimated by using the nephelometry technique. Subjects with urinary albumin levels below 30 mg/g of creatinine were included in the normoalbuminuria group (Group A). Subjects with urinary albumin between 30-300 mg/g of creatinine were included in the microalbuminuria group (Group B) and those with urinary albumin more than 300 mg/g of creatinine were included in the macroalbuminuria group (Group C).

Urinary nephrin estimation was done using an ELISA kit (supplied by ELabscience) with a reported sensitivity of 0.10 ng/mL and a detection range of 0.16-10 ng/mL. For estimation of urinary nephrin levels, values were read in a microplate reader and were calculated with interpolation from the standard curve.

Statistical analysis

Data analysis was done using IBM Corp. Released 2010. IBM SPSS Statistics for Windows, Version 19.0. Armonk, NY: IBM Corp. The distribution of categorical variables such as gender and age of the study subjects were expressed as frequencies and percentages. The distribution of categorical variables among various groups was analyzed using chi-square or Fischer exact test.

Data such as duration of diabetes, urinary albumin, and urinary nephrin were expressed as median with inter-quartile range (IQR). Comparison of urinary nephrin levels between the different groups was done using the Kruskal-Wallis test. Post-hoc analysis between the sub-groups among diabetic subjects was done by Mann-Whitney U test. Correlation between urinary albumin and nephrin levels was done with the help of Spearman’s correlation test.

All statistical analysis was carried out at a 5% level of significance and a p-value of less than 0.05 was considered significant.

## Results

A total of 147 subjects were recruited in this study. The baseline characteristics of the study population are shown in Table 1. It was observed that there was a gradual elevation in serum urea and creatinine levels, proportional to albuminuria among the study subjects. The differences were statistically significant by unpaired student’s t-test for both parameters (Table 1).

**Table 1:** Baseline characteristics of the study population IQR - Interquartile range; SD - Standard deviation

Parameter	Diabetic subjects with normoalbuminuria (Group A) (n = 65)	Diabetic subjects with microalbuminuria (Group B) (n = 45)	Diabetic subjects with macroalbuminuria (Group C) (n = 37)	p-value
Mean age in years (min-max)	52.0 (29-85)	52.8 (30-70)	53.9 (27-86)	-
Male (%)	29 (44.6)	28 (62.2)	18 (48.6)	-
Female (%)	36 (55.4)	17 (37.8)	19 (51.4)	-
Mean height (cm)	155.0	153.5	154.7	-
Mean weight (kg)	65.0	62.7	64.3	-
BMI (kg/m²)	27.3 (17.4-34.9)	26.8 (18.3-37)	26.9 (18.4-39.5)	0.731
Median duration of diabetes in years (IQR)	5 (2-7)	5 (2-8)	4 (2-7)	0.970
Serum urea in mg% (Mean ± SD)	24.5 ± 9.5	26.9 ± 11.9	31.2 ± 11.7	0.020
Serum creatinine in mg% (Mean ± SD)	0.68 ± 0.23	0.72 ± 0.22	0.83 ± 0.25	0.018

Diabetic nephropathy among study subjects was categorized based on urinary albumin values. The subjects were divided into three groups-normoalbuminuria, microalbuminuria and, macroalbuminuria-according to urinary albumin values. Since the data was following a non-normal distribution, the median with IQR for subjects in each of the three categories is given in Table 2.

**Table 2:** Urinary albumin values in the study groups SD - Standard deviation; IQR - Interquartile range

Urinary albumin values in the study groups (mg/g creatinine)			
Groups	N = 147	Mean (SD)	Median (IQR)
Diabetic with normoalbuminuria	65	12.2 (2.4)	10.7 (10.7-13.2)
Diabetic with microalbuminuria	45	57.7 (21.4)	52.8 (40.6-72)
Diabetic with macroalbuminuria	37	444.5 (126.3)	412 (356-521)

Urinary nephrin values were also following a non-normal distribution and hence are being described as median with IQR for the study subjects. It was found that subjects in the microalbuminuria group had a higher median urinary nephrin level than those in the normoalbuminuria and macroalbuminuria groups. The differences in urinary nephrin levels between the three groups were found to be statistically significant using the Kruskal Wallis test (Table 3).

**Table 3:** Urinary nephrin values in the study groups IQR - Interquartile range

Urinary nephrin values in the study groups (ng/mL)			
Group	Median (IQR)	Minimum	Maximum
Diabetic with normoalbuminuria (n = 65)	6.1 (3.3-11.5)	0.1	25.9
Diabetic with microalbuminuria (n = 45)	13.8 (6.3-22.7)	0.3	97.8
Diabetic with macroalbuminuria (n = 37)	11.9 (8.2-18.1)	0.2	82.9
p-value	< 0.001		

The correlation of the study subjects' urinary albumin and nephrin values showed a positive correlation with Spearman’s rho value being 0.356 and p-value below 0.001. There was a weak positive correlation between these two parameters which, was statistically significant.

## Discussion

Diabetic nephropathy is a leading cause of the end-stage renal disease (ESRD). Urinary albumin estimation is the gold standard for the diagnosis of diabetic nephropathy. Type 2 diabetic patients need to be screened for nephropathy by assessing urinary albumin at the time of diagnosis of diabetes.

Recent studies have shown that podocyte-specific proteins like podocin and nephrin were present in the urine of diabetic patients before the appearance of albuminuria. These urinary markers may correlate with the temporal progression of diabetic nephropathy.

Nephrin is a component of the slit-diaphragm in glomeruli that is essential for maintaining the selective permeability of urinary proteins. Nephrin is an integral part of podocytes and along with the basement membrane, and endothelial cells, forms the glomerular filtration barrier. Injury to podocytes leads to the presence of nephrin in urine [5]. In diabetic nephropathy, there is reduced expression of nephrin along with podocytopenia and podocyturia. In a study done by Jim et al., nephrinuria was present in 56% of patients with normoalbuminuria and in all patients with micro and macroalbuminuria [6]. In another study, there was a reduction in the expression of nephrin in renal tubules in type 2 diabetic patients. This impaired the ability of podocytes to recover after injury and rendered them susceptible to detachment [7]. In our study, the median urinary nephrin values were higher for patients with micro and macroalbuminuria than for those with normoalbuminuria. These findings were similar to those in studies done by Jim et al., and Patari et al. [6, 8]. However, urinary nephrin values were more elevated in patients with microalbuminuria than in those with macroalbuminuria. The cause for the disproportionate elevation of urinary nephrin values in patients in these two groups (microalbuminuria & macroalbuminuria) was not clear.

Urinary albumin was not detected in 60% of subjects with normoalbuminuria. However, urinary nephrin was seen in these subjects. This finding suggests that nephrinuria can occur before the onset of albuminuria in diabetic subjects. Similar results were reported by Jim et al., in their study where 56% of diabetic patients with normoalbuminuria had nephrinuria.

Our study showed no significant difference between urinary nephrin and urinary albumin on correlation analysis. This suggests that urinary nephrin excretion increases with a rise in urinary albumin excretion in diabetic patients. There was no significant difference in urinary nephrin levels among diabetic patients with microalbuminuria and macroalbuminuria in our study. This finding was in contrast to increased urinary nephrin levels in patients with advanced stages of diabetic nephropathy reported in other studies. Patari et al., in their study, had found similar levels of urinary nephrin in patients in all stages of diabetic nephropathy. However, urinary nephrin level was analyzed by the western blot technique in their study [8].

In a study done by Daniel PK Ng et al., nephrinuria was independently associated with the logarithmic form of an albumin-creatinine ratio (ACR) and estimated glomerular filtration rate (eGFR). Nephrinuria was associated with these traits in type 2 diabetic patients with normoalbuminuria [9]. Another study also showed an association between biomarkers of podocyte damage like urinary nephrin and vascular endothelial growth factor with normoalbuminuria in patients with type 2 diabetes mellitus [10]. Urinary nephrin was found to be a sensitive and specific marker for diagnosing early diabetic nephropathy than microalbumin in another study [11].

Since this was a cross-sectional study, a temporal relationship between urinary nephrin levels and the onset of diabetic nephropathy could not be established. Diabetic subjects with reduced glomerular filtration rate (GFR) were not included in this study. Hence, a definite relationship of urinary nephrin levels with GFR could not be ascertained. These could be considered as limitations of the study.

## Conclusions

Urinary nephrin levels are increased in patients with type 2 diabetic nephropathy as compared to those without nephropathy. Urinary nephrin level was more elevated in diabetic patients with microalbuminuria than in those with normoalbuminuria and macroalbuminuria. Urinary nephrin level has a weak positive correlation with albuminuria in patients with type 2 diabetic nephropathy.
